# Experimental and numerical investigations of arc plasma expansion in an industrial vacuum arc remelting (VAR) process

**DOI:** 10.1038/s41598-022-24595-7

**Published:** 2022-11-27

**Authors:** Ebrahim Karimi-Sibaki, Mario Peyha, Alexander Vakhrushev, Menghuai Wu, Andreas Ludwig, Jan Bohacek, Bernhard Preiss, Abdellah Kharicha

**Affiliations:** 1 Christian-Doppler Laboratory for Metallurgical Applications of Magnetohydrodynamics, Montanuniversitaet of Leoben, Franz-Josef-Str. 18, 8700 Leoben, Austria; 2 Chair of Simulation and Modeling of Metallurgical Processes, Montanuniversitaet of Leoben, Franz-Josef-Str. 18, 8700 Leoben, Austria; 3 Chair of Process Technology and Industrial Environmental Protection, Montanuniversitaet of Leoben, Franz-Josef-Str. 18, 8700 Leoben, Austria; 4 Heat Transfer and Fluid Flow Laboratory, Faculty of Mechanical Engineering, Brno University of Technology, Technicka 2896/2, 616 69 Brno, Czech Republic; 5 INTECO Melting and Casting Technologies GmbH, 8600 Bruck an der Mur, Austria

**Keywords:** Mechanical engineering, Computational methods, Plasma physics

## Abstract

In the present study, we investigate arc plasma expansion in an industrial vacuum arc remelting (VAR) process using experimental and numerical tools. Stainless steel is the alloy of interest for the electrode (cathode) and ingot (anode). During the operation of the VAR process, behaviors of cathode spots and plasma arc were captured using the high-speed camera (Phantom v2512). We found that spots prefer to onset and remain within the partially melted surface at the center of the electrode tip. Existing spots outside the melting zone accelerate toward the edge of the electrode to extinguish. We observed a fairly symmetrical and centric plasma column during the operation. For further investigation of the observed arc column in our experiment, we used the two-fluid magnetohydrodynamics (MHD) model of plasma proposed by Braginskii. Thus, we modeled the arc column as a mixture of two continuous interpenetrating compressible fluids involving ions and electrons. Through numerical simulations, we calculated plasma parameters such as number density of ions/electrons, electric current density, flow of ions/electrons, temperature of ions/electrons, and light intensity for the observed arc column in our experiment. The calculated light intensity of plasma was compared with images captured by the camera to verify the model. The distribution of electric current density along the surface of the anode, namely ingot, is a decisive parameter that impacts the quality of the final product (ingot) in VAR process. Herein, we confirm that the traditionally used Gaussian distribution of electric current density along the surface of the ingot is viable.

## Introduction

**Figure 1:**
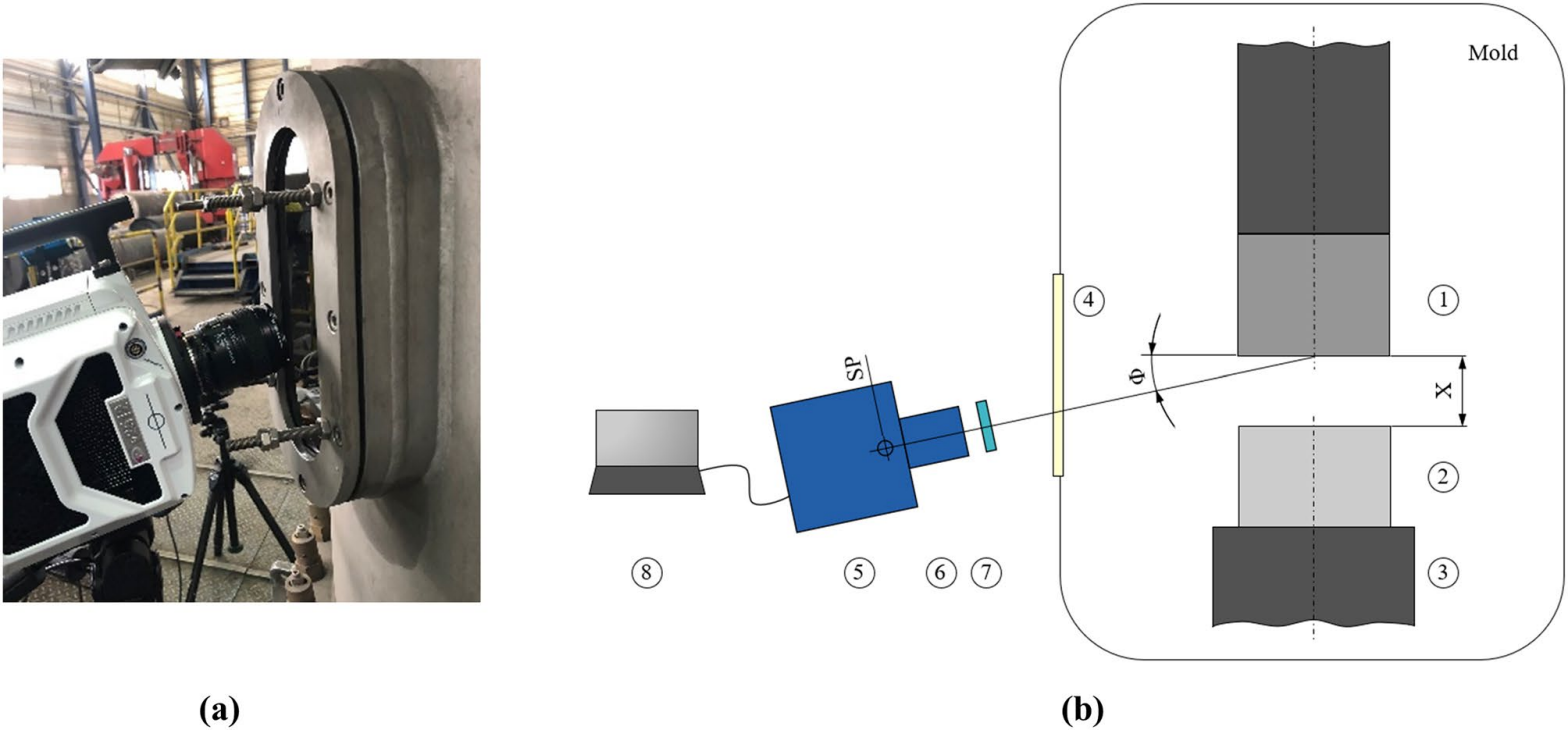
(**a**) The high-speed camera (Phantom v2512) near the furnace to visualize the process, (**b**) a schematic representation of the experimental setup including 1—electrode, 2—ingot, 3—base plate, 4—inspection glass, 5—high-speed camera, 6—lens, 7—circular polarizing filter, 8—computer with camera control software. X, ϕ, and SP denote the gap, the camera angle to electrode surface plane, and sensor plane, respectively.

Diverse application areas exist for vacuum arcs in vacuum interrupters, vacuum deposition, vacuum thrusters, and vacuum arc remelting (VAR) processes^1,2^. VAR, as a secondary metallurgical process, is employed to manufacture ultra-clean alloys such as titanium-based, nickel-based, and stainless steel. The impure alloy called electrode in VAR remelts with the aid of an electric arc under vacuum condition, as shown schematically in Fig. 1. The forming droplets at the tip of the consumable electrode pass through the vacuum to reach the melt pool. The melt pool solidifies in a water-cooled mold to build the chemically homogenous and clean ingot. Contaminants such as Pb, Sn, Bi, Cu and low-density oxides are evaporated or transferred to the exterior surface of the ingot near the mold.

The electric arc, which provides the thermal energy to remelt the electrode, is maintained between the tip of the melting electrode (cathode) and the top of the ingot (anode). The existence of arc is dependent on cathode spots which move in an erratic way over the surface of the cathode^2–7^. The arc, emanated from the spots, comprises ions and electrons. The arc, always operating in the DC mode, carries an enormous amount of electric current in the range of 5–40 kA. It is established knowledge that the arc plays a decisive role in the overall energy transfer to the top of ingot, the flow in the melt pool, and consequently, the quality of the final ingot^8–10^. Thus, forecasting the arc distribution in the vacuum region and on the ingot top (anode) is of great importance.

Research groups conducted several experiments to study the behavior of cathode spots on the melting electrode^6,11^ and the movement and distribution of the arc^6,12–14^ in the vacuum region of VAR process. Tezenas du Montcel et al.^6^ pointed out that the distribution of cathode spots on the surface of the electrode and the distribution of the arc in the vacuum region are interdependent. Considering distinct cathode spot distributions, they observed three different arc modes: diffuse, diffuse columnar, and multiple arcs. Chapelle et al.^11^ investigated the influences of cathode spots on mechanisms of transfer of the liquid metal from the tip of the electrode to the top of the ingot, including growth, rupture, and erosion of metal drop during VAR of a Titanium alloy. Also, they evaluated the parameters of the cathode spots, including their current, size, and apparent velocity on the melting electrode.

The behavior of vacuum arc plasma has been subject to many studies^1,2^. An early experiment conducted by Schellekens^15^ aimed to determine the characteristic quantities of the plasma, like ion/electron temperature, for the diffuse vacuum arc. Woodside et al.^12^ performed an experiment to determine the arc distribution in an industrial VAR process for the production of Ti64 alloy. They used a noninvasive measurement system involving externally mounted magnetic flux density sensors. Based on magnetic field measurement around the VAR furnace, they reported three patterns for the arc, including diffusive, constricted eccentric, and rotating arcs. Delzant et al.^13^ placed photodiodes inside a VAR furnace of Ti64 alloy to evaluate arc movement based on the analysis of the light emitted by the arc. They reported three patterns, including a constricted centric arc, a back-and-forth motion of the arc centroid between two stable off-centred areas, and a back-and-forth motion of the arc following an elliptic path. Cibula et al.^14^ proposed using a transverse magnetic field for active control of the arc position and arc movement in VAR. They recorded images and measured arc luminosity during VAR of a titanium alloy. Three arc distributions were reported, including diffusive, constricted, and semi-constricted composed of multiple arc columns^14^.

Numerical simulations are globally accepted as a valuable tool to get insight into the behavior of high current vacuum arcs (HCVA), especially in a vacuum circuit breaker^16^. Assuming an isothermal plasma, Keider et al.^17^ and Beilis et al.^18^ proposed a 2D MHD model of plasma by combining the fluid equations with the electromagnetic equations. Schade and Shmelev^19^ extended the MHD model to include the energy conservation of electrons and ions. Wang et al.^20^ and Zhang et al.^21^ considered the ion viscosity and used the Biot-Savart law to extensively study the effect of several nonhomogeneous distributions of the axial magnetic field (AMF) on the expansion of plasma. Langlois et al.^22^ and Tezenas du Montcel et al.^23^ modeled a diffused arc considering the possibility of both supersonic (at low current density) and subsonic (at high current density) flow regimes of plasma under the action of AMF in the vacuum circuit breaker.

Chapelle et al.^10^ modeled the plasma generation and expansion in the VAR process. They simplify the problem by assuming a constant ratio of the electron to the ion velocity to remove the transport equation of magnetic field from MHD equations of plasma^10^. The distribution of electric current density along the surface of the anode, which has a significant impact on the final quality of the ingot (anode) in VAR, was calculated^10^. Conventionally, a Gaussian distribution is employed to model the arc distribution at the ingot top by introducing a parameter, namely arc radius (fraction of ingot radius)^24–29^. The arc radius is unknown a priori. Several simulation trials must be performed to estimate the proper arc radius, which was the subject of many investigations^24–29^. The adequacy of this implicit way of modeling arc through Gaussian distribution is scrutinized in this paper.

We employ the full two-fluid MHD model of plasma, including the transport equation of magnetic field^20,22^. Simulation input parameters such as geometrical configuration and operation parameters are based on the settings of the experiment, which we recently performed in collaboration with Breitenfeld Edelstahl AG in Austria using an industrial VAR process. The aforementioned experimental studies^6,11–14^ were performed using titanium-based, zirconium-based, or copper-based alloys. In the present study, the electrode (cathode) and ingot (anode) are made of stainless steel, which to the best of our knowledge, has never been used for the experimental investigation in the VAR process. Herein, the behavior of cathode spots on the electrode is only experimentally studied. The expansion of arc plasma in the vacuum region is investigated both experimentally and numerically. A comparison is made between our experimental and numerical results to verify the model. The main goal is to obtain some fundamental understanding of the plasma generation and expansion in an industrial VAR process.

## Methods

### Experiment

We performed the experiment using a 300 mm diameter electrode (cathode) and a 300 mm ingot (anode) in an industrial VAR furnace. This standard full-scale VAR furnace is located in the melt shop of Breitenfeld Edelstahl AG, Austria. Both electrode and ingot were made of stainless steel. The vacuum chamber, namely mold, of the VAR furnace was made of copper with a diameter of 1050 mm. Right after arc initiation, the electrode was lifted upward by the control system of the VAR furnace to create a stable gap, distance between the anode and cathode, with the size of 40 mm. The magnitude of electric current flowing through the system, also known as arc current, was 5 kA. The duration of the experiment was limited to a few tens of seconds to prevent the full melting of the tip of the electrode (cathode).

The armhole with a mounted inspection glass enabled optical accessibility to the interior of the furnace, especially to the electrode tip. A high-speed camera (Phantom v2512) was used to visualize the process, as shown in Fig. 1a. The optical setup also involves a camera lens (Nikon AF Micro Nikkor 60 mm 1:2,8D), a circular polarizing filter (Hoya PL-CIR 62 mm) and a computer with the camera control software (PCC 3.4). Due to the light emitted by the process itself, no external light source was used. Considering the gap size (X ≈ 40 mm), the free working distance of 800 mm, and an inclination angle to the electrode front plane (ϕ ≈ 12° ± 1°), the field of view was set to 896 × 400 pixels (≈ 349 mm × 156 mm) during the experiment as schematically shown in Fig. 1b. These settings enabled a recording rate of 60,000 frames per second (60 k fps) for all videos. During the recording, camera settings were fine-tuned to a lens aperture of f/8, an exposure time of 3 µs and a high dynamic range (HDR) of 0.8 µs.

### Modeling

#### Governing equations

**Figure 2:**
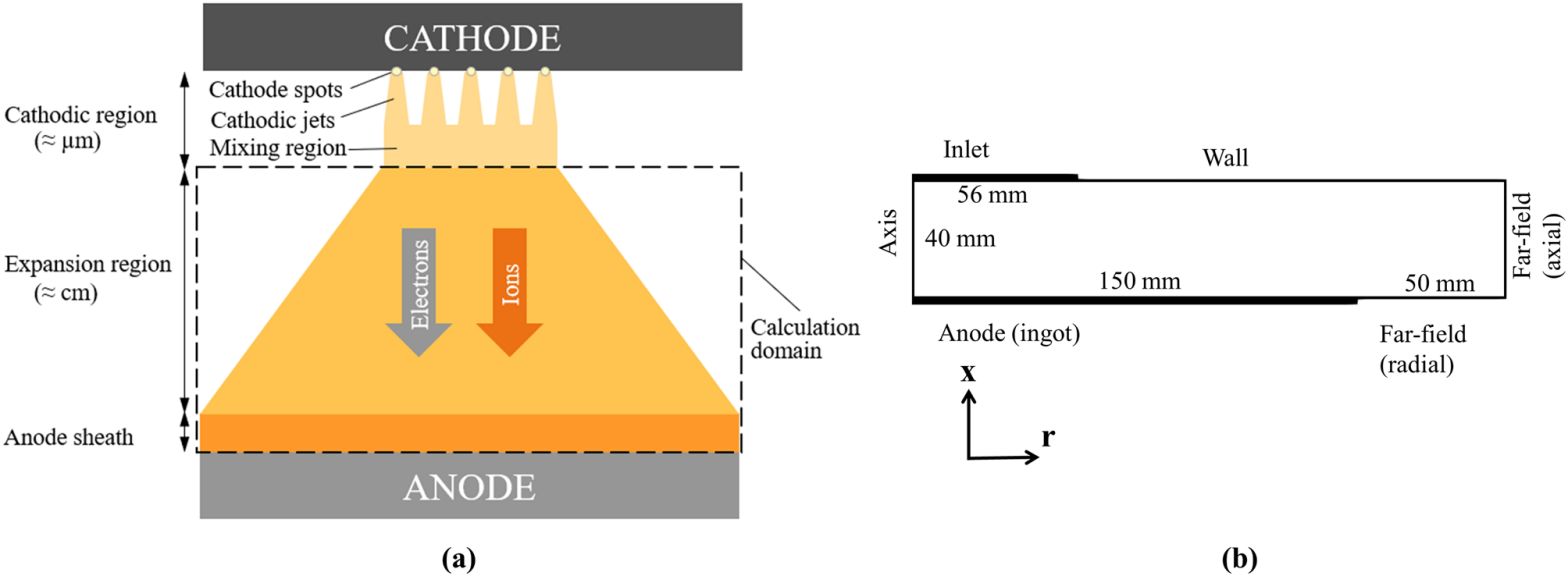
(**a**) Schematic representation of the diffuse vacuum arc, (**b**) schematic representation of the computational domain including dimensions and boundaries.

According to the classical vacuum arc theory, the diffuse vacuum arc, schematically shown in Fig. 2a, can be divided into distinct regions^22,23^: the cathodic plasma jets emanating from each cathode spot, the jet mixing region, the expansion region, which forms the main body of the arc column, and the anode sheath. Herein, our modeling activities are focused on the arc column and anode sheath. The calculation domain is marked using the dashed rectangle shown in Fig. 2a. The following assumptions are made:

1. Following Chapelle et al.^5,10^, the length of the cathodic region (~ μm), shown in Fig. 2a, is negligible compared to that of the expansion region (~ cm). Thus, the exit of the cathodic region, which is the entry of the expansion region, hereinafter referred to as “Inlet”, is located right below the cathode. We ignore the length of the cathodic region in our calculations, and we assume that the gap length is 40 mm, as reported in our experiment. The diameter of the Inlet differs from that of the cathodic electrode. During the experiment, as explained in more detail later, we observed that the majority of cathode spots appeared in the partially melted zone at the center of the electrode tip. Thus, we assume that the area of the Inlet is equivalent to that of the melted zone. All plasma parameters distribute uniformly, and velocities are normal to the boundary at the Inlet^5,10,19,22^.
2. During the experiment, as explained in more detail later, we observed a fairly centric, symmetrical arc. We assume that the self-induced magnetic field generated by the arc is dominantly in the azimuthal direction, and the computational domain is 2D axisymmetric. The computational domain, including dimensions and boundaries, is shown in Fig. 2b.
3. The anode is in the passive state and generates no secondary plasma. The anode sheath is implicitly modeled considering special boundary conditions for electric potential, magnetic field, and electron energy conservation^19,21,22,30,31^. They will be further elucidated after introducing governing equations.
4. The arc column (plasma) features the following characteristics^10,16,19,22^: plasma reaches a steady-state condition; electron inertia is ignored as the mass of electron is several orders of magnitudes smaller than the mass of ions; plasma is fully ionized involving only ions and electrons with an unchanged average charge (${\mathrm{Z}}_{\mathrm{i}}$); plasma remains quasi-neutral (${\mathrm{n}}_{\mathrm{e}}={\mathrm{Z}}_{\mathrm{i}}{\mathrm{n}}_{\mathrm{i}}$); electron fluid and ion fluid are considered as ideal gases.

The magneto hydrodynamics (MHD) approach is used to describe the plasma^10,16,19,22,32^.

1 $$\nabla \cdot {(\mathrm{n}}_{\mathrm{i}}{\mathbf{u}}_{\mathbf{i}})=0$$

2 $$\nabla \cdot {(\mathrm{n}}_{\mathrm{i}}{\mathrm{m}}_{\mathrm{i}}{\mathbf{u}}_{\mathbf{i}}{\mathbf{u}}_{\mathbf{i}})=-\nabla ({\mathrm{P}}_{\mathrm{e}}+{\mathrm{P}}_{\mathrm{i}})+\nabla \cdot {(\widehat{\uptau }}_{\mathrm{i}})+\mathbf{j}\times \mathbf{B}$$

3 $${\mathbf{u}}_{\mathbf{e}}={\mathbf{u}}_{\mathbf{i}}-\frac{\mathbf{j}}{{\mathrm{n}}_{\mathrm{e}}\mathrm{e}}$$

4 $$\nabla \cdot \left(-\frac{1}{{\upmu }_{0}\upsigma }\nabla {\mathbf{B}}_{\uptheta }\right)=\nabla \times {\left({\mathbf{u}}_{\mathbf{e}}\times \mathbf{B}\right)}_{\uptheta }+\frac{1}{{{\mathrm{n}}_{\mathrm{e}}}^{2}\mathrm{e}}{\left(\nabla {\mathrm{P}}_{\mathrm{e}}\times \nabla {\mathrm{n}}_{\mathrm{e}}\right)}_{\uptheta }-\frac{1}{{\upsigma }^{2}}{\left(\mathbf{j}\times \nabla\upsigma \right)}_{\uptheta }+\frac{1}{{\upmu }_{0}{\upsigma }^{2}}\left(\nabla {\mathbf{B}}_{\uptheta }\cdot \nabla\upsigma \right)- \frac{{\mathbf{B}}_{\uptheta }}{{\upmu }_{0}\upsigma {\mathrm{r}}^{2}}$$

5 $$\frac{3}{2}{\mathrm{n}}_{\mathrm{i}}\mathrm{k }{\mathbf{u}}_{\mathbf{i}}\cdot \nabla {\mathrm{T}}_{\mathrm{i}} +\nabla \cdot {(\mathrm{P}}_{\mathrm{i}}{\mathbf{u}}_{\mathbf{i}})+{{\mathrm{m}}_{\mathrm{i}}\mathrm{n}}_{\mathrm{i}}{\mathbf{u}}_{\mathbf{i}}\cdot \nabla (\frac{{\mathbf{u}}_{\mathbf{i}}^{2}}{2}) =\nabla \cdot ({\uplambda }_{\mathbf{i}}\nabla {\mathrm{T}}_{\mathrm{i}})+\nabla \cdot {(\widehat{\uptau }}_{\mathrm{i}}\cdot {\mathbf{u}}_{\mathbf{i}})+{\mathrm{Q}}_{\mathrm{i}}$$

6 $$\frac{3}{2}{\mathrm{n}}_{\mathrm{e}}\mathrm{k }{\mathbf{u}}_{\mathbf{e}}\cdot \nabla {\mathrm{T}}_{\mathrm{e}} +\nabla \cdot {(\mathrm{P}}_{\mathrm{e}}{\mathbf{u}}_{\mathbf{e}})+{{\mathrm{m}}_{\mathrm{e}}\mathrm{n}}_{\mathrm{e}}{\mathbf{u}}_{\mathbf{e}}\cdot \nabla \text{(}\frac{{\mathbf{u}}_{\mathbf{e}}^{2}}{2}\text{) }=\nabla \cdot ({\uplambda }_{\mathbf{e}}\nabla \, {\mathrm{T}}_{\mathrm{e}}+\frac{{\mathrm{C}}_{1}\mathrm{k}}{\mathrm{e}}\mathbf{j}{\mathrm{T}}_{\mathrm{e}})+{\mathrm{Q}}_{\mathrm{e}}$$

Mass conservation, Eq. (1), and momentum conservation, Eq. (2), of ions are calculated to determine the flow field. In Eq. (2), Lorentz force, ${\mathbf{F}}_{\mathbf{L}}=\mathbf{j}\times \mathbf{B}$, and viscous stress, ${\widehat{\uptau }}_{\mathrm{i}} ={\upmu }_{\mathrm{i}}\left[\left(\nabla {\mathbf{u}}_{\mathbf{i}}+\nabla {\mathbf{u}}_{\mathbf{i}}^{\mathbf{T}}\right)-\frac{2}{3}\nabla \cdot {\mathbf{u}}_{\mathbf{i}}\mathbf{I}\right]$, are considered where ion viscosity, ${\upmu }_{\mathrm{i}}=\frac{0.406{\left(4\uppi {\upvarepsilon }_{0}\right)}^{2}\sqrt{{\mathrm{m}}_{\mathrm{i}} }}{{{\mathrm{Z}}_{\mathrm{i}}}^{4}{\mathrm{e}}^{4}\mathrm{ln\Lambda }}{({\mathrm{kT}}_{\mathrm{i}})}^\frac{5}{2}$, and elastic collision between ions and electrons through Coulomb logarithm, $\mathrm{ln\Lambda }\approx 15.93+\mathrm{ln}\left(\frac{{\mathrm{T}}_{\mathrm{e}}^\frac{3}{2}}{{\mathrm{n}}_{\mathrm{e}}^\frac{1}{2}}\right)$, are taken into account. Additionally, ion pressure, ${\mathrm{P}}_{\mathrm{i}}={\mathrm{n}}_{\mathrm{i}}{\mathrm{kT}}_{\mathrm{i}}$, and electron pressure, ${\mathrm{P}}_{\mathrm{e}}={\mathrm{n}}_{\mathrm{e}}{\mathrm{kT}}_{\mathrm{e}}$, follow ideal gas law.

The velocity field of electrons is related to the electric current density field and ion velocity field through Eq. (3). As previously mentioned, the magnetic field transport equation, Eq. (4), is solved assuming that the self-induced magnetic field generated by the arc, is dominantly in the azimuthal direction.

In Eq. (4), the electrical conductivity of plasma, $\upsigma =\frac{{\mathrm{C}}_{3}{\mathrm{n}}_{\mathrm{e}}{\mathrm{e}}^{2}}{{\mathrm{m}}_{\mathrm{e}}{\upupsilon }_{\mathrm{ei}}}$, is dependent on the collision frequency, ${\upupsilon }_{\mathrm{ei}}=\frac{{{{\mathrm{n}}_{\mathrm{e}}\mathrm{ Z}}_{\mathrm{i}}}^{2}{\mathrm{e}}^{4}\mathrm{ln\Lambda }}{3 \sqrt{{\mathrm{m}}_{\mathrm{e}} }{{\upvarepsilon }_{0}}^{2}{(2\uppi {\mathrm{kT}}_{\mathrm{e}})}^{1.5}}$ , between ions and electrons. Radial component of electric current density, ${\mathrm{j}}_{\mathrm{r}}=-\frac{1}{{\upmu }_{0}}\frac{\partial {\mathbf{B}}_{\uptheta }}{\partial \mathrm{x}}$, and axial component of electric current density, ${\mathrm{j}}_{\mathrm{x}}=\frac{1}{{\upmu }_{0}}(\frac{\partial {\mathbf{B}}_{\uptheta }}{\partial \mathrm{r}}+\frac{{\mathbf{B}}_{\uptheta }}{\mathrm{r}})$, are easily obtained through Ampere’s law. The induced electric current density in the tangential direction due to the electron Hall effect is negligible as no external magnetic field is considered.

The energy conservation equation for ions, Eq. (5), is solved to determine the ion thermal field. Thermal conductivity of ions, ${{{{\uplambda }_{\mathrm{i}}=\mathrm{C}}_{0}\uplambda }_{\mathrm{e}}(\frac{{\mathrm{T}}_{\mathrm{i}}}{{\mathrm{T}}_{\mathrm{e}}})}^\frac{5}{2}{(\frac{{\mathrm{m}}_{\mathrm{e}}}{{\mathrm{m}}_{\mathrm{i}}})}^\frac{1}{2}$, and energy exchange between ions and electrons through collision, ${\mathrm{Q}}_{\mathrm{i}}=\frac{3{\mathrm{m}}_{\mathrm{e}}}{{\mathrm{m}}_{\mathrm{i}}}{\mathrm{n}}_{\mathrm{e}}{\upupsilon }_{\mathrm{ei}}\mathrm{k}{(\mathrm{T}}_{\mathrm{e}}-{\mathrm{T}}_{\mathrm{i}})$, are considered.

The energy conservation equation for electrons, Eq. (6), is solved to attain the electron thermal field. Thermal conductivity of electrons, ${\uplambda }_{\mathrm{e}}=\frac{{\mathrm{C}}_{2}{\mathrm{k}}^{2}{\mathrm{n}}_{\mathrm{e}}{\mathrm{T}}_{\mathrm{e}}}{{\mathrm{m}}_{\mathrm{e}}{\upupsilon }_{\mathrm{ei}}}$, and thermal source term, ${\mathrm{Q}}_{\mathrm{e}}=-{\mathrm{Q}}_{\mathrm{i}}+\frac{{\mathbf{j}}^{2}}{\upsigma }+\frac{{\mathrm{C}}_{1}\mathrm{k}}{\mathrm{e}}\mathbf{j}\cdot \nabla {\mathrm{T}}_{\mathrm{e}}$, are regarded. The latter is dependent on energy exchange between ions and electrons through collision, Joule heating ($\frac{{\mathbf{j}}^{2}}{\upsigma }$), and electron thermal work ($\frac{{\mathrm{C}}_{1}\mathrm{k}}{\mathrm{e}}\mathbf{j}\cdot \nabla {\mathrm{T}}_{\mathrm{e}}$).

Of note, constant coefficients like ${\mathrm{C}}_{0}$, ${\mathrm{C}}_{1}$, ${\mathrm{C}}_{2}$, ${\mathrm{C}}_{3}$ were advised to be related to the charge average number^22^. Herein, we use standard values for those coefficients reported in Ref.^21^.

#### Boundary conditions

The 2D axisymmetric computational domain, including dimensions and boundaries, is illustrated in Fig. 2b. Considering that the cathode and anode are made of stainless steel in the experiment, a major encountered challenge is the lack of sufficient and reliable input parameters for the plasma. Before describing boundary conditions, a caveat is in order concerning input parameters, in particular, at the Inlet. The calculations were carried out as a first approximation using the values reported for the simulation of high current vacuum arcs (HCVA) as input parameters. A more rigorous approach would be desirable in a future work to check the validity of these approximated values for plasma parameters at the Inlet.

Following Wang et al.^31^, Zhang et al.^21^, and Han et al.^30^, the velocity normal to the surface is about 10^3^ m s^−1^; ion temperature is about 10 eV; electron temperature is about 1.5 eV. Those values are considered to be uniformly distributed over the Inlet. Ion number density at the Inlet can be obtained through: ${{\mathrm{n}}_{\mathrm{i}}{\mathrm{m}}_{\mathrm{i}}\mathrm{ u}}_{\mathrm{ix}}=\frac{{\mathrm{\mu I}}_{0}}{\uppi {\mathrm{R}}_{\mathrm{c}}^{2}}$ where the value of about $100\mathrm{ \mu g }{\mathrm{C}}^{-1}$ is assumed for the erosion rate (μ)^21,30,31,33^. To complete all necessary boundary conditions for the Inlet, it is assumed that the electric current density also distributes uniformly^21,30,31^. Thus, the magnitude of the azimuthal magnetic field is set to ${\mathrm{B}}_{\uptheta }=\frac{{\upmu }_{0}{\mathrm{I}}_{0}}{2\uppi {\mathrm{R}}_{\mathrm{c}}^{2}}\mathrm{r}$.

At the Wall, a condition of zero flux for ion, Eq. (5), and electron, Eq. (6), energy equations are assigned where slip boundary condition for the flow of ions is considered. The magnitude of the azimuthal magnetic field is set to ${\mathrm{B}}_{\uptheta }=\frac{{\upmu }_{0}{\mathrm{I}}_{0}}{2\mathrm{\pi r}}$.

At the Anode, the static pressure is fixed to the critical value, which is the pressure for the Mach number equal to one^30^. Special care must be taken to include implicitly the anode sheath in the magnetic and energy transports of the arc. For that purpose, the potential drop in the anode sheath, ${\mathrm{\varphi }}_{\mathrm{sh}}=\frac{{\mathrm{kT}}_{\mathrm{e}}}{\mathrm{e}}\mathrm{ln}\left(\frac{{\mathrm{u}}_{\mathrm{ex}}}{0.25{\mathrm{u}}_{\mathrm{e},\mathrm{th}}}\right)$, and electron thermal speed, ${\mathrm{u}}_{\mathrm{e},\mathrm{th}} =\sqrt{\frac{8{\mathrm{kT}}_{\mathrm{e}}}{\uppi {\mathrm{m}}_{\mathrm{e}}}}$, are defined^19,21,22,30,31^. The total flux of electron energy, Eq. (6), is: ${{\mathrm{n}}_{\mathrm{e}}\mathrm{e u}}_{\mathrm{ex}}\left(\frac{2{\mathrm{kT}}_{\mathrm{e}}}{\mathrm{e}}-{\mathrm{\varphi }}_{\mathrm{sh}}\right)$. The total flux of the azimuthal magnetic field is assigned as follows^19,21,22,30,31^:

7 $$-\frac{1}{{\upmu }_{0}\upsigma }\left(\frac{\partial {\mathbf{B}}_{\uptheta }}{\partial \mathrm{z}}\right)=-{\mathrm{u}}_{\mathrm{ex}}{\mathbf{B}}_{\uptheta }+\frac{1}{{\mathrm{n}}_{\mathrm{e}}\mathrm{e}}\frac{\partial {\mathrm{P}}_{\mathrm{e}}}{\partial \mathrm{r}}+\frac{{\mathrm{C}}_{1}\mathrm{k}}{\mathrm{e}}\frac{\partial {\mathrm{T}}_{\mathrm{e}}}{\partial \mathrm{r}}+\frac{\partial {\mathrm{\varphi }}_{\mathrm{sh}}}{\partial \mathrm{r}}$$

At the Far-Field boundaries, conditions are determined by exploiting the fact that the expansion of the plasma arc in the vacuum leads to the rapid decrease in the ion density, accompanied by an acceleration and cooling of ions, and consequently falling of total plasma pressure^10,19,21,22,31^. It is evaluated that the pressure may fall several orders of magnitude even if the arc plasma travels a short distance (~ 1 cm) between electrodes^10,19,21,22,31^. Indeed, the Far-Field boundaries, as the interfaces between the plasma and vacuum ambience, are dummy boundaries. Ensuring continuity of plasma parameters, and considering that free-stream conditions exist at Far-field boundaries, the pressure-far-field boundary condition is employed^34^. Thereby, the Mach number (here tantamount to one as plasma is compressible), exit velocity direction (radial or axial), and the pressure are specified. As the pressure is unknown, three different values were examined, including 10 Pa, 20 Pa, and 30 Pa. The influences of those selected pressures on the calculated field structures, such as ion/electron temperature, electric current density, and flow fields, are examined. They will be further discussed in “Discussions”.

The system is enclosed by the Far-Field (axial), where the magnetic field is set to ${\mathrm{B}}_{\uptheta }=\frac{{\upmu }_{0}{\mathrm{I}}_{0}}{2\mathrm{\pi r}}$. To achieve continuity of magnetic field through the Far-Field (radial), Eq. (7) without the sheath term ($\frac{\partial {\mathrm{\varphi }}_{\mathrm{sh}}}{\partial \mathrm{r}}$), is used to assign the flux of magnetic field. In a similar manner to disregard sheath term at Far-Field boundaries, the total flux of electron energy at the Far-Field (radial), ${{\mathrm{n}}_{\mathrm{e}}\mathrm{e u}}_{\mathrm{ex}}\left(\frac{2{\mathrm{kT}}_{\mathrm{e}}}{\mathrm{e}}\right)$, and at the Far-Field (axial),${{\mathrm{n}}_{\mathrm{e}}\mathrm{e u}}_{\mathrm{er}}\left(\frac{2{\mathrm{kT}}_{\mathrm{e}}}{\mathrm{e}}\right)$, are specified.

At the axis, only the magnitude of the magnetic field is set to zero. Zero flux is considered for all other parameters.

#### Other settings

All governing equations of flow, electromagnetic, and ion/electron energy conservation are discretized according to the well-established Finite Volume Method^34^. Several user-defined-functions (UDF) are implemented into the commercial software, ANSYS FLUENT v.17.1, to model arc characteristics (e.g., electrical conductivity, collision frequency, etc.), boundary conditions (e.g., anode sheath, etc.) and transport equations (e.g., ion/electron energy, magnetic field).

All geometrical dimensions are shown in Fig. 2b. It is recalled that the distance among electrodes is 40 mm, and the radius of the computational domain is 200 mm. Quadrilateral square-shaped mesh elements with the size of 1 mm are generated to fill the computational domain with a total number of 40 × 200 = 8000 cells. Simulation trials were performed to verify that additional mesh refinement has no significant influence on the simulation results. All parameters used in our calculations are listed in Table 1.

**Table 1:** Parameters used in our calculations.

Parameter	Unit	Value
${\mathrm{C}}_{0}$		1.0
${\mathrm{C}}_{1}$		1.5
${\mathrm{C}}_{2}$		2.5
${\mathrm{C}}_{3}$		1
$\mathrm{e}$	$\mathrm{C}$	1.609 × 10^–19^
${\mathrm{I}}_{0}$	$\mathrm{kA}$	5
$\mathrm{k}$	${\mathrm{m}}^{2}\mathrm{ kg }{\mathrm{s}}^{-2} {\mathrm{K}}^{-1}$	1.3806 × 10^–23^
$\mathrm{L}$	$\mathrm{mm}$	40
${\mathrm{m}}_{\mathrm{i}}$	$\mathrm{kg}$	9.273 × 10^–26^
${\mathrm{m}}_{\mathrm{e}}$	$\mathrm{kg}$	9.109 × 10^–31^
${\mathrm{R}}_{\mathrm{A}}$	$\mathrm{mm}$	150
${\mathrm{R}}_{\mathrm{C}}$	$\mathrm{mm}$	56
${\mathrm{Z}}_{\mathrm{i}}$		1.8
$\upgamma$		1.666
$\upmu$	$\mathrm{\mu g }{\mathrm{C}}^{-1}$	100
${\upmu }_{0}$	$\mathrm{H }{\mathrm{m}}^{-1}$	4π × 10^–7^
${\upvarepsilon }_{0}$	$\mathrm{F }{\mathrm{m}}^{-1}$	8.854 × 10^–12^

## Results

### Experimental results

**Figure 3:**
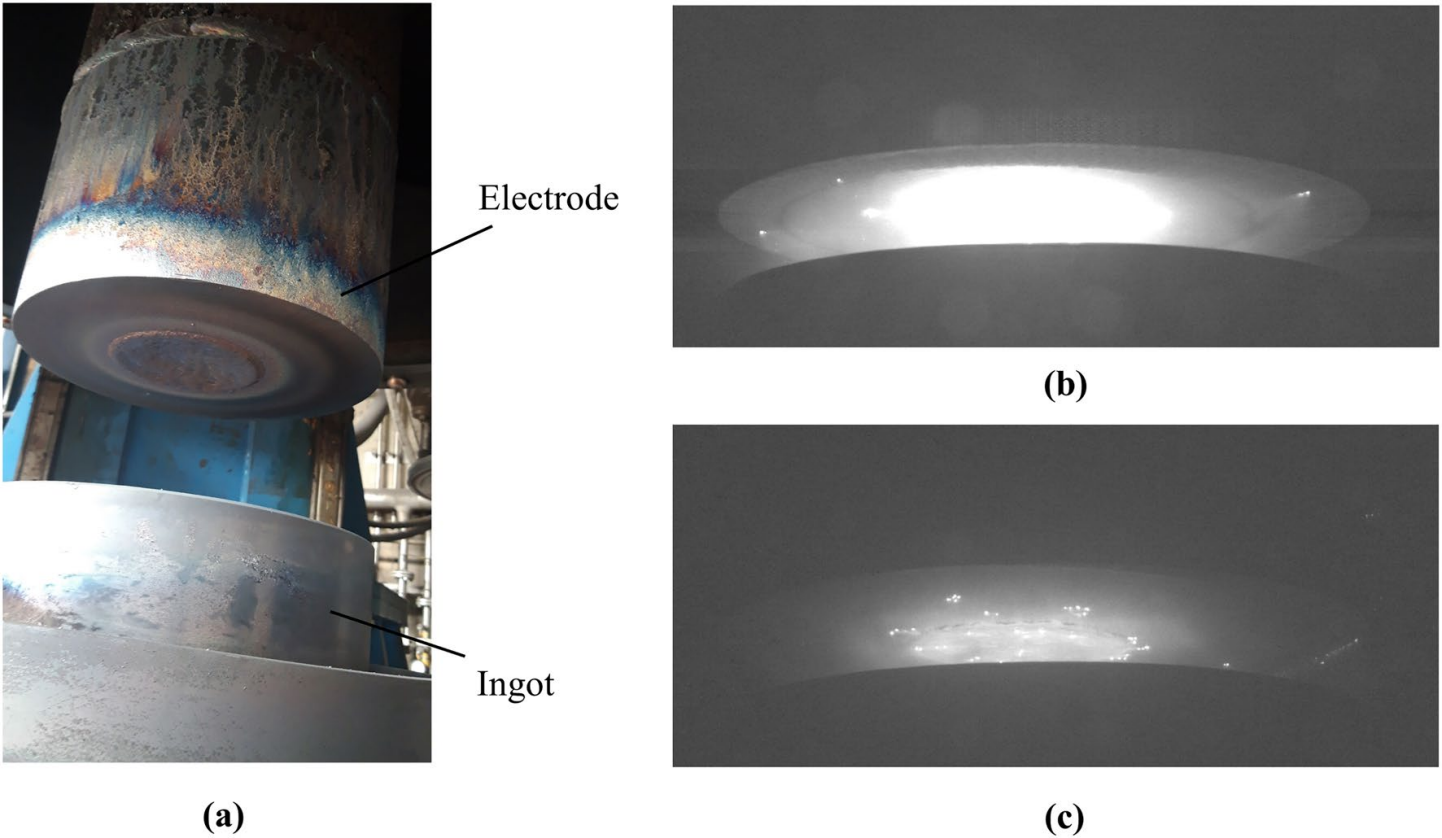
(**a**) Demonstration of electrode and ingot right after performing the experiment, (**b**) experimental demonstration of arc plasma, (**c**) experimental demonstration of cathode spots.

As previously mentioned, both electrode (cathode) and ingot (anode) were made of stainless steel in our experiment. Figure 3a shows the electrode and ingot right after performing the experiment. An intense light was detected around the center of the gap between the electrode and ingot, indicating the expansion of plasma, as shown in Fig. 3b. The display settings (e.g. gain, brightness, contrast, etc.) for our recorded HDR images provide a qualitative visual impression of the plasma. On the other hand, melting the central part of electrode, as shown in Fig. 3a, altered the lighting conditions of the process. This, in turn, enabled us to capture sharp images of cathode spots, as exemplary shown in Fig. 3c.

It is not possible to effectively demonstrate the behaviors of cathode spots or arc plasma using images, although some snapshots of different times are shown in this section. Therefore, we highly encourage readers to observe supplemental materials of this paper, including “Videos S1.avi” and “Videos S2.avi”.

**Figure 4:**
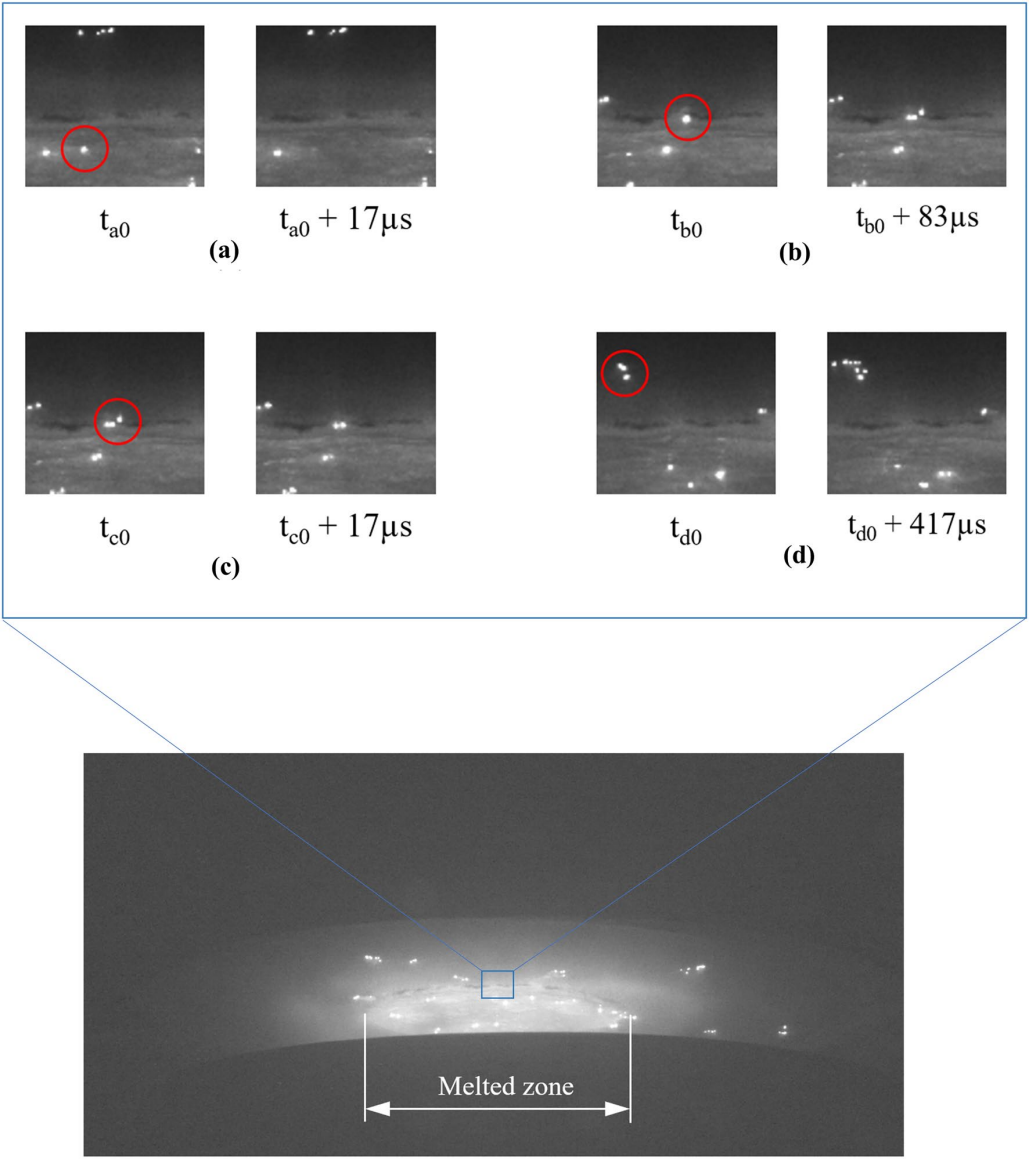
Snapshots at different times at a zoomed area on the cathode to indicate cathode spots behavior. The red circle is used to spotlight each observed phenomenon. (**a**) The cathode spot disappears within the melted zone, (**b**) the cathode spot splits at the phase boundary, (**c**) the cathode spot disappears at the phase boundary, (**d**) cathode spots split and form a cluster at the solid part of the cathode.

The arc is emanated from cathode spots which move in an erratic way over the surface of the cathode. Cathode spots have a finite lifetime, while the arc expands within the vacuum zone. Therefore, they extinguish and reform on the cathode surface. The general tendency is that spots prefer to onset at the melted zone, and they less frequently appear on the solid part of the cathode. We observed distinct phenomena associated with behavior of cathode spots during our experiment. A summary is shown in Fig. 4. Of note, t_a0_, t_b0_, t_c0_, and t_d0_ are instants during our camera recording related to each observed phenomenon in Fig. 4a–d, respectively. Some spots disappear within the melted zone, as shown in Fig. 4a. The survived cathode spots within the melt rush toward the phase boundary, where they stagnate and split to create additional spots, as shown in Fig. 4b. Most often, those spots stagnate at the phase boundary, where they later disappear, as shown in Fig. 4c. Occasionally, emerged spots on the solid part of the cathode form a group (clustering) while accelerating toward the edge of the electrode, as shown in Fig. 4d.

Despite the ever-changing locations of the cathode spots, the arc column remains in a quasi-steady state, as demonstrated in the supplemental material “Videos S2.avi”. Although the arc oscillates while expanding in the vacuum, the arc column remains moderately centric and symmetrical, as exemplary shown in Fig. 3b.

### Numerical results

As mentioned in “Boundary conditions”, the pressure is unknown at Far-field boundaries. This unknown parameter is subject to a parametric study. For that purpose, we examined three different values, including 10 Pa, 20 Pa, and 30 Pa. All results are presented in the Supplementary information document, namely “All_Computed_Field_Structures.pdf”. Herein, results concerning the distributions of the main plasma parameters, as an example, are presented for the case with the limiting pressure of 20 Pa at Far-field boundaries.

**Figure 5:**
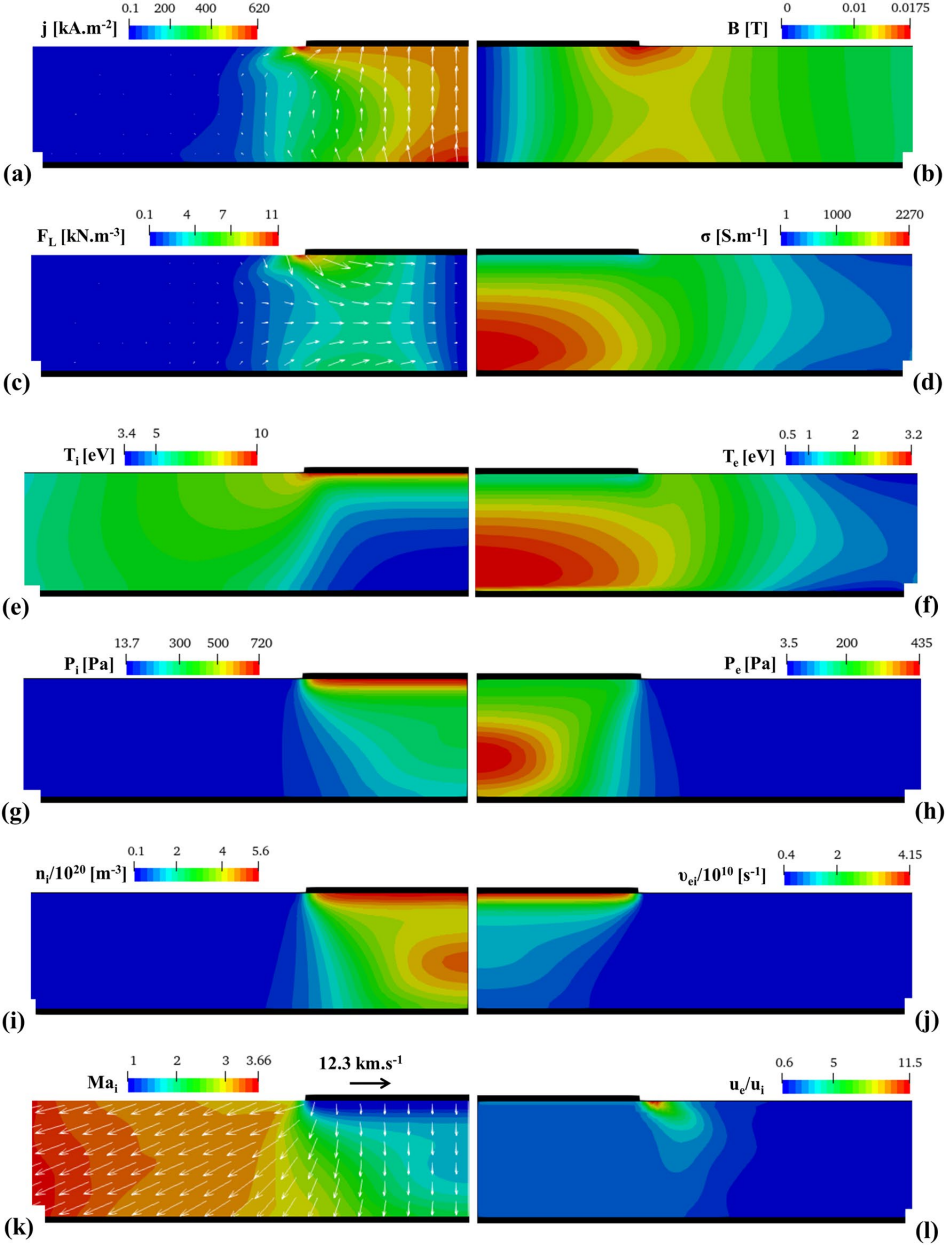
Field structures including (**a**) electric current density, (**b**) magnetic flux density, (**c**) Lorentz force, (**d**) electrical conductivity, (**e**) ion temperature, (**f**) electron temperature, (**g**) ion pressure, (**h**) electron pressure, (**i**) ion number density, (**j**) collision frequency, (**k**) ion Mach number, (**l**) ratio of electron to ion velocity.

The distribution of electric current density is shown in Fig. 5a. A notable amount of electric current density flows near the edge of Inlet. The electric current density contracts near the center of the Anode due to the sheath effect. The presence of anode sheath impacts the radial electric field through the term ($\frac{\partial {\mathrm{\varphi }}_{\mathrm{sh}}}{\partial \mathrm{r}}$) that, in turn, enforces the electric current constriction. The magnetic field, shown in Fig. 5b, is directly correlated to the electric current density through Ampere’s law. Thus, regions of intense magnetic fields are predicted near the edge of Inlet and in the vicinity of the mid-radius of the anode. The magnetic field remains minimal near the axis. The distribution of Lorentz force is shown in Fig. 5c. Lorentz force is originated in the interaction between electric current density and magnetic field through ${\mathbf{F}}_{\mathbf{L}}=\mathbf{j}\times \mathbf{B}$. Thus, the peak value of Lorentz force is near the edge of Inlet, where both magnetic field and electric current density are strong. Globally, Lorentz force is directed toward the axis except near the edge of the Inlet, where it bends downward. As shown in Fig. 5d, the electrical conductivity of plasma experiences peak values above the central part of the Anode.

The ion temperature, as shown in Fig. 5e, drops sharply in the central part of the arc near the axis as a consequence of the rapid expansion of plasma in the vacuum. The peak of ion temperature is under the shadow of the Inlet. As shown in Fig. 5f, peak values of electron temperature are above the central part of the Anode under the shadow of the Inlet. This is attributed to electric current constriction, the elevation of Joule heating ($\frac{{\mathbf{j}}^{2}}{\upsigma }$), and consequently, heat generation in electrons. The electron temperature drops in front of the anode surface, which is caused by the electron crossing the anode sheath and consequently losing thermal energy^22,30^. Ion pressure, as shown in Fig. 5g, significantly drops from the Inlet to the anode due to the rapid expansion of plasma. The pressure field of the electron, as shown in Fig. 5h, resembles the thermal field of the electron, as the peak value is above the Anode axis. Following ideal gas law to determine pressure fields of ions and electrons, the pressures of those entities are associated with temperature and number density.

Both ions and electrons undergo a strong expansion. Therefore, a significant decrease in the number density is predicted from the axis toward the edge of the Anode, as shown in Fig. 5i. Peak values of ion number density are at two areas: (1) a constricted high density near the Inlet, (2) in the central area of the arc column where ions/electrons encounter the notable Lorentz force exerted through the electric current density constriction. Collision frequency, as shown in Fig. 5j, remains potent near the Inlet. Collision frequency gradually decreases from the Inlet toward the Anode as plasma expands in the vacuum. Ion velocity vectors and contour of Ion Mach number (${\mathrm{Ma}}_{\mathrm{i}} =\frac{\Vert {\mathbf{u}}_{\mathbf{i}}\Vert }{\sqrt{\frac{\mathrm{\gamma k}{\mathrm{T}}_{\mathrm{i}}}{{\mathrm{m}}_{\mathrm{i}}}}}$) are overlaid, as shown in Fig. 5k. The acceleration of ions during the plasma expansion is responsible for the increase of ion Mach number. The Mach number exceeds 3.5 near the edge of the anode, corresponding to the ion velocity greater than 10^4^ m s^−1^. The supersonic ion flow regime during the arc expansion in the vacuum, also predicted in the present study, has been reported in literatures^19,21,22,31,35^.

Finally, the ratio of electron to ion velocity is shown in Fig. 5l. The ratio is an important parameter which enables us to develop reduced order models (ROMs) for vacuum arc plasma expansion. Such a model was occasionally used considering a constant ratio (e.g. 20.04) inside the entire vacuum region to simplify governing equations^10^. Using the ratio allows the removal of the transport equation of magnetic field from MHD equations, that in turn helps us to obtain a rough first approximation of plasma distribution^10^. As shown in Fig. 5l, The ratio remains relatively constant under the shadow of Inlet. However, the variation of this ratio from the edge of the Inlet toward the edge of the Anode is no longer negligible.

## Discussions

As previously mentioned, plasma pressure at Far-field boundaries is unknown a priori. Therefore, we performed several simulations considering the fact that the plasma pressure drops several orders of magnitude during the expansion in the vacuum. For that purpose, we scrutinized three different limiting values of plasma pressure, including 10, 20, and 30 Pa. All results are presented in the Supplementary information document, namely “All_Computed_Field_Structures.pdf”.

**Figure 6:**
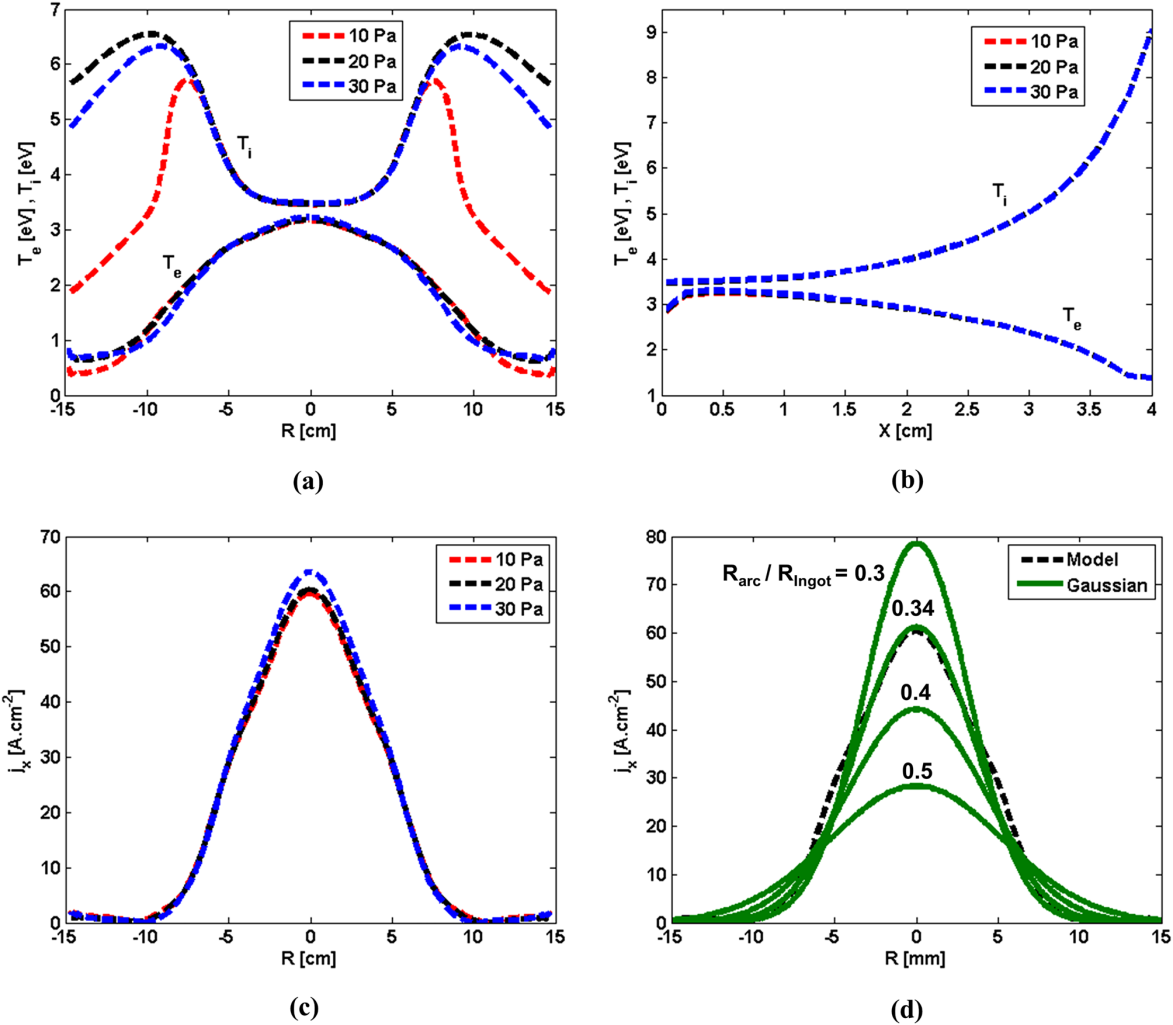
(**a**) Ion temperature and electron temperature along the surface of Anode, (**b**) ion temperature and electron temperature along the axis of symmetry, (**c**) electric current density along the surface of Anode, (**d**) a comparison between the calculated electric current density and prescribed Gaussian distribution along the surface of Anode.

Our simulation trials revealed that the patterns observed for different field structures, shown in Fig. 5, remained unchanged. However, a slight shift in the magnitude and locations of those parameters was reckoned. For the sake of demonstration, variations of decisive parameters such as ion temperature, electron temperature, and axial current density along the axis and Anode surface are extracted. The ion temperature and electron temperature along the surface of the Anode are plotted as shown in Fig. 6a. The ion temperature is higher than the electron temperature for all investigated pressures. Results remain identical under the shadow of Inlet with a radius of ca. 8 cm. This radius is notably larger than the radius of Inlet. Although the discrepancy between calculated results for the electron temperature remains minimal, notable deviations in the calculated ion pressure are observed near the edge of the Anode. The discrepancies drop as the selected pressure increases. The temperatures are plotted along the axis of the domain, as shown in Fig. 6b. The ion temperature always exceeds the electron temperature. Results remain identical regardless of the pressures.

The axial current density, as shown in Fig. 6c, determines the amount of electric current which is transmitted to the Anode surface. A significant constriction of the arc exists. Thus, a substantial amount of the electric current density is collected near the axis. The collected amount of electric current density rapidly decreases as the distance from the symmetry axis increases. As shown in Fig. 6c, the selected limiting pressures appear to have a slight influence on the distribution of electric current density on the Anode surface.

Indeed, the axial current density (especially at Anode) is a pivotal parameter in evaluating the performance of industrial high current vacuum arc (HCVA) processes such as vacuum interrupters^16,36,37^, and vacuum arc remelting (VAR)^10,24–27,29^. Most often, for the sake of simplicity and to avoid extra complexity related to the modeling of arc plasma, the distribution of electric current density over the anode is specified using the Gaussian function^24–27,29,36,37^. Afterwards, the investigation focuses on the behavior of liquid metal to explore the anode melting pool (AMP), which, for example, determines the quality of the final product in VAR. Herein, the validity of the Gaussian function as a reduced order model (ROM) is examined, as shown in Fig. 6d. The Gaussian function, ${\mathrm{j}}_{\mathrm{x}}=\frac{{\mathrm{I}}_{0}\mathrm{exp}(-\frac{{\mathrm{r}}^{2}}{{\mathrm{R}}_{\mathrm{arc}}^{2}})}{{\int }_{0}^{{\mathrm{R}}_{\mathrm{ingot}}}2\mathrm{\pi rexp}(-\frac{{\mathrm{r}}^{2}}{{\mathrm{R}}_{\mathrm{arc}}^{2}})\mathrm{ dr}}$, requires a prescribed value as known as arc radius, ${\mathrm{R}}_{\mathrm{arc}}$, that is customarily a fraction of ingot radius^24–27,29,38^. As shown in Fig. 6d, the Gaussian function, employing the arc radius equivalent to 0.34 times of the ingot radius, reasonably agrees with the model results that indicate the viability of this simplifying and practical approach.

Shang et al.^39^ proposed a method to calculate the intensity of the light emitted from the arc aiming at the validation of numerical results using images of video records of the arc. They made the following assumptions: (1) the plasma is optically thin so that the light absorption is neglected, (2) the path of view is perpendicular to the electrode plane, (3) The light intensity along the path of view, $\mathrm{I }(\mathrm{y})$, is proportional to the number density of ions. Thus, the light intensity is expressed as^39^:

8 $$\mathrm{I }\left(\mathrm{y}\right)\approx {\int }_{\mathrm{y}}^{{\mathrm{R}}_{\mathrm{A}}}\frac{{\mathrm{n}}_{\mathrm{i}}\left(\mathrm{r}\right)\mathrm{r}}{\sqrt{{\mathrm{r}}^{2}-{\mathrm{y}}^{2}}}\mathrm{ dr}$$

**Figure 7:**
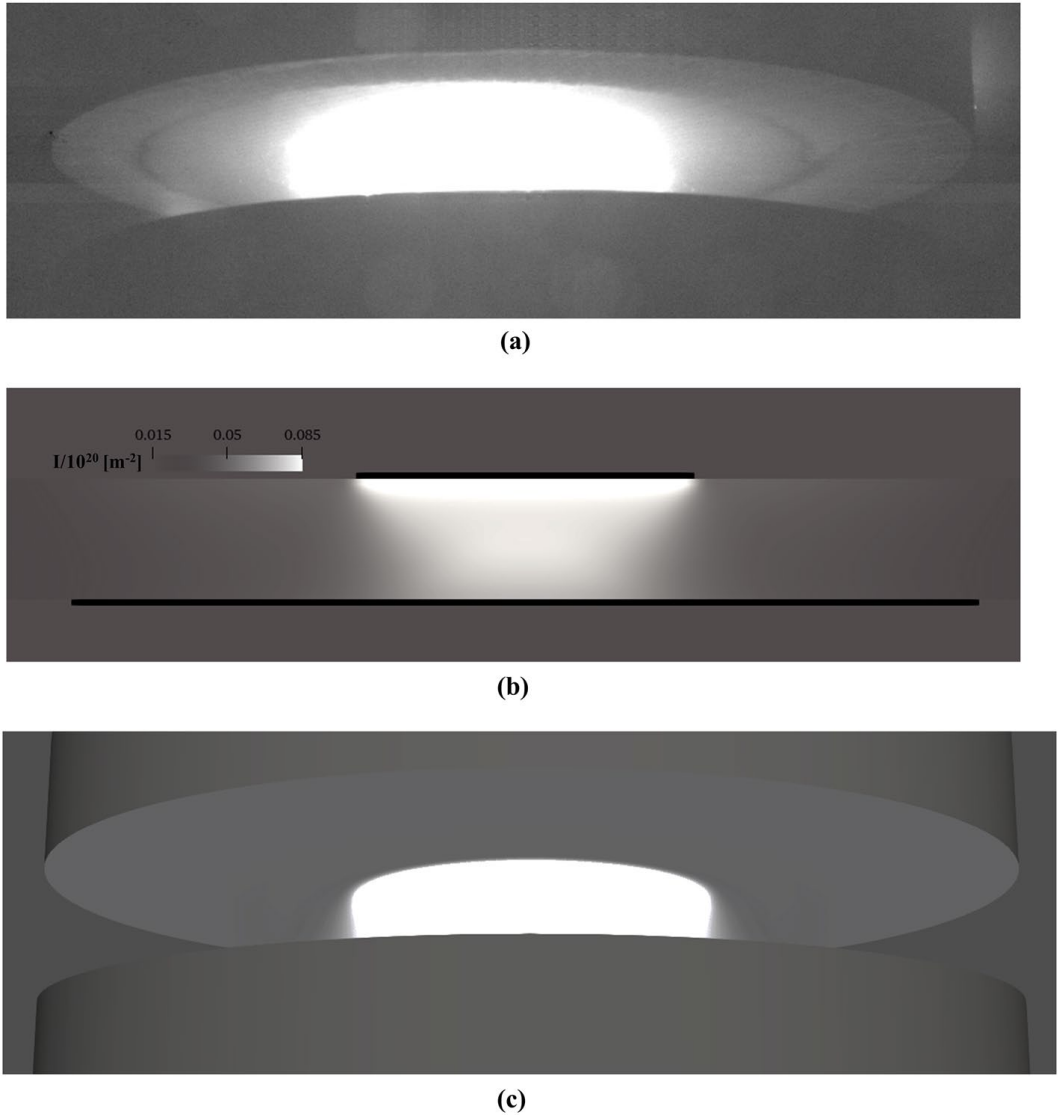
(**a**) Experimental arc, (**b**) calculated light intensity, (**c**) reconstructed 3D arc.

The experimental arc and the calculated arc through Eq. (8) are shown in Fig. 7. The highest intensity appears in the arc center near the Inlet. The constriction of the arc column toward the Anode can be observed, as shown in Fig. 7b. By rotation of the horizontal slices of the 2D model results, a 3D representation of the simulated arc is reconstructed, as shown in Fig. 7c. Following the experimental setup, a camera is positioned 12° below the rendered scene, including a light source aligned with the view line in the postprocessor (ParaView v.5.10). It is rather complex to apply the same optical settings of the experiment during the postprocessing as the glowing of the arc due to the radiation could not be reconstructed. However, a good qualitative agreement can be observed considering the experimental and the 3D reconstructed arc.

## Summary

We performed an experiment, in collaboration with Breitenfeld Edelstahl AG in Austria, using a high-speed camera (Phantom v2512) to study cathode spots dynamics and plasma arc expansion in an industrial vacuum arc remelting (VAR) process. The cathode (electrode) and anode (ingot) were made of stainless steel. The behavior of cathode spots on the electrode was only experimentally studied. We observed that most of spots prefer to onset and remain within the partially melted electrode area. Occasionally, a few spots, onset outside of the melting zone, accelerate toward the edge of electrode in the retrograde direction to extinct.

The expansion of arc plasma in the vacuum region was investigated both experimentally and numerically. We observed a fairly centric and symmetrical arc during the experiment. The arc plasma was modeled using the magnetohydrodynamics (MHD) approach. Considering a 2D axisymmetric model, simulation input parameters such as geometrical configuration and operation parameters were based on the settings of our experiment. Plasma parameters such as electric current density, magnetic field, Lorentz force, electrical conductivity, ion/electron temperature, ion/electron pressure, number density, ion velocity/Mach number, and the ratio of electron to ion velocity were computed.

The distribution of plasma parameters (ions/electrons temperature, electric current density) on the anode (ingot) surface are of great importance. Based on calculation results, we confirm the viability of the traditionally used Gaussian distribution to specify electric current density distribution along the surface of the anode (ingot). The light emitted from the plasma is reconstructed to compute the light intensity. The method integrates the calculated number density of ions along the viewing path. We verified the modeling results by comparing the calculated light intensity with images of video records of the arc.

## Supplementary Information

Supplementary Information.

## Data Availability

The datasets used and/or analyzed during the current study are available from the corresponding author upon reasonable request.

## Abbreviations

$\mathbf{B}$: Magnetic field vector, $\mathrm{T}$
${\mathbf{B}}_{\uptheta }$: Azimuthal magnetic field, $\mathrm{T}$
${\mathrm{C}}_{0}$: Constant coefficient for ion thermal conductivity
${C}_{1}$: Constant coefficient for electron thermal work
${\mathrm{C}}_{2}$: Constant coefficient for electron thermal conductivity
${\mathrm{C}}_{3}$: Constant coefficient for plasma electrical conductivity
$\mathrm{e}$: Absolute value of electron charge, $\mathrm{C}$
${\mathbf{F}}_{\mathrm{L}}$: Volumetric Lorentz force vector, $\mathrm{N }{\mathrm{m}}^{-3}$
$$\mathrm{I}$$: Light intensity, $${\mathrm{m}}^{-2}$$
${\mathrm{I}}_{0}$: Imposed electrical current, $\mathrm{A}$
$$\mathbf{I}$$: Identity matrix
$\mathbf{j}$: Electric current density vector, $\mathrm{A }{\mathrm{m}}^{-2}$
${\mathrm{j}}_{\mathrm{r}}$: Radial current density, $\mathrm{A }{\mathrm{m}}^{-2}$
${\mathrm{j}}_{\mathrm{x}}$: Axial current density, $\mathrm{A }{\mathrm{m}}^{-2}$
$$\mathrm{k}$$: Boltzmann constant, $${\mathrm{m}}^{2}\mathrm{ kg }{\mathrm{s}}^{-2} {\mathrm{K}}^{-1}$$
$L$: Electrode gap length, $\mathrm{m}$
$${\mathrm{m}}_{\mathrm{i}}$$: Ion mass, $$\mathrm{kg}$$
$${\mathrm{m}}_{\mathrm{e}}$$: Electron mass, $$\mathrm{kg}$$
${Ma}_{i}$: Ion Mach number
${Ma}_{e}$: Electron Mach number
${\mathrm{n}}_{\mathrm{i}}$: Number density of ions, ${\mathrm{m}}^{-3}$
${\mathrm{n}}_{\mathrm{e}}$: Number density of electrons, ${\mathrm{m}}^{-3}$
$${\mathrm{P}}_{\mathrm{i}}$$: Ion pressure, $$\mathrm{Pa}$$
$${\mathrm{P}}_{\mathrm{e}}$$: Electron pressure, $$\mathrm{Pa}$$
${\mathrm{Q}}_{\mathrm{i}}$: Electron–ion energy exchange, $\mathrm{W }{\mathrm{m}}^{-3}$
${\mathrm{Q}}_{\mathrm{e}}$: Total electron energy source, $\mathrm{W }{\mathrm{m}}^{-3}$
${\mathrm{q}}_{\mathrm{ez}}^{*}$: Total electron energy flux at anode, $\mathrm{W }{\mathrm{m}}^{-2}$
$${\mathrm{R}}_{\mathrm{A}}$$: Anode radius, $$\mathrm{m}$$
$${\mathrm{R}}_{\mathrm{arc}}$$: Arc radius, $$\mathrm{m}$$
$${\mathrm{R}}_{\mathrm{ingot}}$$: Ingot radius, $$\mathrm{m}$$
$${\mathrm{R}}_{\mathrm{C}}$$: Cathode radius, $$\mathrm{m}$$
$$\mathrm{r}$$: Radial coordinate, $$\mathrm{m}$$
$$\mathrm{x}$$: Axial coordinate, $$\mathrm{m}$$
$${\mathrm{T}}_{\mathrm{i}}$$: Ion temperature, $$\mathrm{K}$$
$${\mathrm{T}}_{\mathrm{e}}$$: Electron temperature, $$\mathrm{K}$$
${\mathbf{u}}_{\mathbf{i}}$: Ion velocity vector, $\mathrm{m }{\mathrm{s}}^{-1}$
${\mathrm{u}}_{\mathrm{ix}}$: Axial ion velocity, $\mathrm{m }{\mathrm{s}}^{-1}$
${\mathrm{u}}_{\mathrm{ir}}$: Radial ion velocity, $\mathrm{m }{\mathrm{s}}^{-1}$
${\mathbf{u}}_{\mathbf{e}}$: Electron velocity vector, $\mathrm{m }{\mathrm{s}}^{-1}$
${\mathrm{u}}_{\mathrm{ex}}$: Axial electron velocity, $\mathrm{m }{\mathrm{s}}^{-1}$
${\mathrm{u}}_{\mathrm{er}}$: Radial electron velocity, $\mathrm{m }{\mathrm{s}}^{-1}$
${\mathrm{u}}_{\mathrm{e},\mathrm{th}}$: Electron thermal velocity, $\mathrm{m }{\mathrm{s}}^{-1}$
$\nabla {\mathbf{u}}_{\mathbf{i}}^{\mathbf{T}}$: Transpose of ion velocity gradient tensor, ${\mathrm{s}}^{-1}$
${\mathrm{Z}}_{\mathrm{i}}$: Average charge of ions
${\uplambda }_{\mathrm{i}}$: Ion thermal conductivity, $\mathrm{W }{\mathrm{K}}^{-1}{\mathrm{m}}^{-1}$
${\uplambda }_{\mathrm{e}}$: Electron thermal conductivity, $\mathrm{W }{\mathrm{K}}^{-1}{\mathrm{m}}^{-1}$
${\widehat{\uptau }}_{\mathrm{i}}$: Ion stress tensor, $\mathrm{N }{\mathrm{m}}^{-2}$
$$\upgamma$$: Adiabatic index
$\upmu$: Cathode erosion rate, $\mathrm{kg }{\mathrm{C}}^{-1}$
$${\upmu }_{\mathrm{i}}$$: Ion viscosity, $$\mathrm{kg }{\mathrm{s}}^{-1}{\mathrm{m}}^{-1}$$
$${\upmu }_{0}$$: Magnetic permeability, $$\mathrm{H }{\mathrm{m}}^{-1}$$
$${\upvarepsilon }_{0}$$: Electric permittivity, $$\mathrm{F }{\mathrm{m}}^{-1}$$
${\mathrm{\varphi }}_{\mathrm{sh}}$: Anode sheath potential drop, $\mathrm{V}$
${\upupsilon }_{\mathrm{ei}}$: Electron–ion collision frequency, ${\mathrm{s}}^{-1}$
$$\mathrm{ln\Lambda }$$: Coulomb logarithm
$$\upsigma$$: Electrical conductivity, $$\mathrm{S }{\mathrm{m}}^{-1}$$
$$\nabla \cdot$$: Divergence operator, $${\mathrm{m}}^{-1}$$
$$\overrightarrow{\nabla }$$: Gradient operator, $${\mathrm{m}}^{-1}$$
