# Supplementary material for: Experimental and numerical investigations of arc plasma expansion in an industrial vacuum arc remelting (VAR) process
Source: Sci Rep. 2022 Nov 27;12:20405. doi: 10.1038/s41598-022-24595-7 (PMC9701783; doi:10.1038/s41598-022-24595-7)
Supplement: Supplementary file 1 — Supplementary Information. [file 41598_2022_24595_MOESM1_ESM.zip › SuppMaterial_revised/All_Computed_Field_Structures.pdf]

Supplementary Information: "All computed field structures"

This document is supplementary information for the paper entitled "Experimental and numerical investigations of arc plasma expansion in an industrial vacuum arc remelting (VAR) process", authored by E. Karimi-Sibaki et al.

As we pointed out in the manuscript, the pressure is unknown at Far-field boundaries. This unknown parameter is subject to a parametric study. For that purpose, we examined three different values, including 10 Pa, 20 Pa, and 30 Pa. Here, the influences of those selected pressures on the calculated field structures are presented. Figure S1 shows field structures considering far-field pressure of 10 Pa. Figure S2 (also presented as Fig 5 in the paper) shows field structures considering far-field pressure of 20 Pa. Figure S3 indicates field structures considering far-field pressure of 30 Pa.

Most fields, such as electric current density, magnetic flux density, Lorentz force, electrical conductivity, electron temperature, ion pressure, electron pressure, ion number density, collision frequency, and the ratio of electron to ion velocity, are slightly influenced by the choice of the above-mentioned pressure. Thus, the distributions of those parameters remain almost unchanged regardless of the assigned far-field pressure.

Ion temperature and, consequently, ion Mach number are parameters which are notably influenced by the magnitude of the assigned far-field pressure. To effectively demonstrate discrepancies, different scales are used to illustrate ion temperature/ion Mach number for different figures. The discrepancy between calculated results for the ion temperature/ion Mach number remains minimal under the shadow of the plasma entry boundary referred to as "Inlet" in the paper. However, notable dissimilarity in the calculated ion temperature/ion Mach number fields is predicted away from the mid-radius of the Anode toward somewhere near the edge of Anode. The discrepancies decrease as the selected pressure increases.

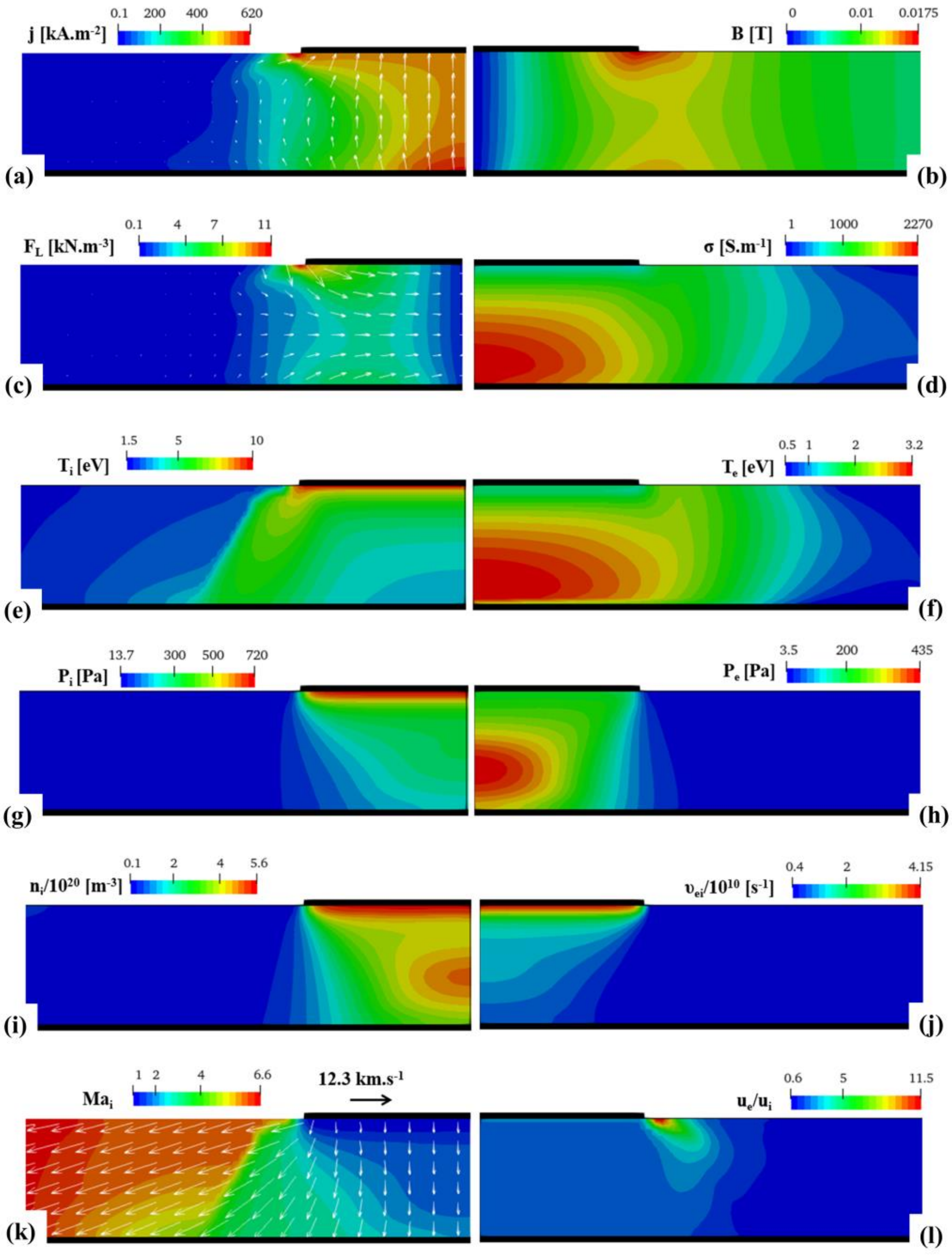

Figure S1: Field structures considering far-field assigned pressure of **10 pa** (a) Electric current density, (b) Magnetic flux density, (c) Lorentz force, (d) Electrical conductivity, (e) Ion temperature, (f) Electron temperature, (g) Ion pressure, (h) Electron pressure, (i) Ion number density, (j) Collision frequency, (k) Ion Mach number, (l) ratio of electron to ion velocity.

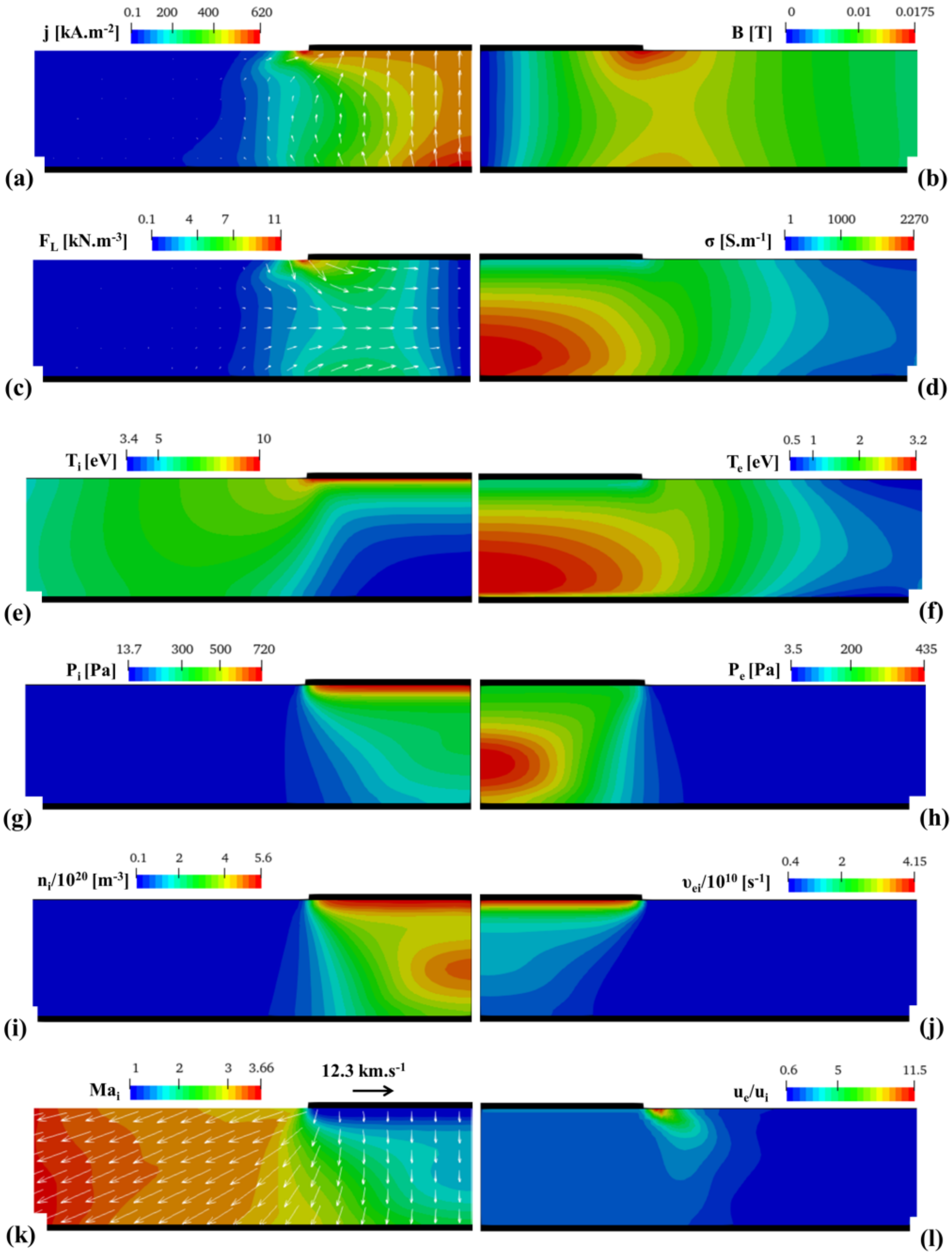

Figure S2: Field structures considering far-field assigned pressure of **20 pa** (a) Electric current density, (b) Magnetic flux density, (c) Lorentz force, (d) Electrical conductivity, (e) Ion temperature, (f) Electron temperature, (g) Ion pressure, (h) Electron pressure, (i) Ion number density, (j) Collision frequency, (k) Ion Mach number, (l) ratio of electron to ion velocity.

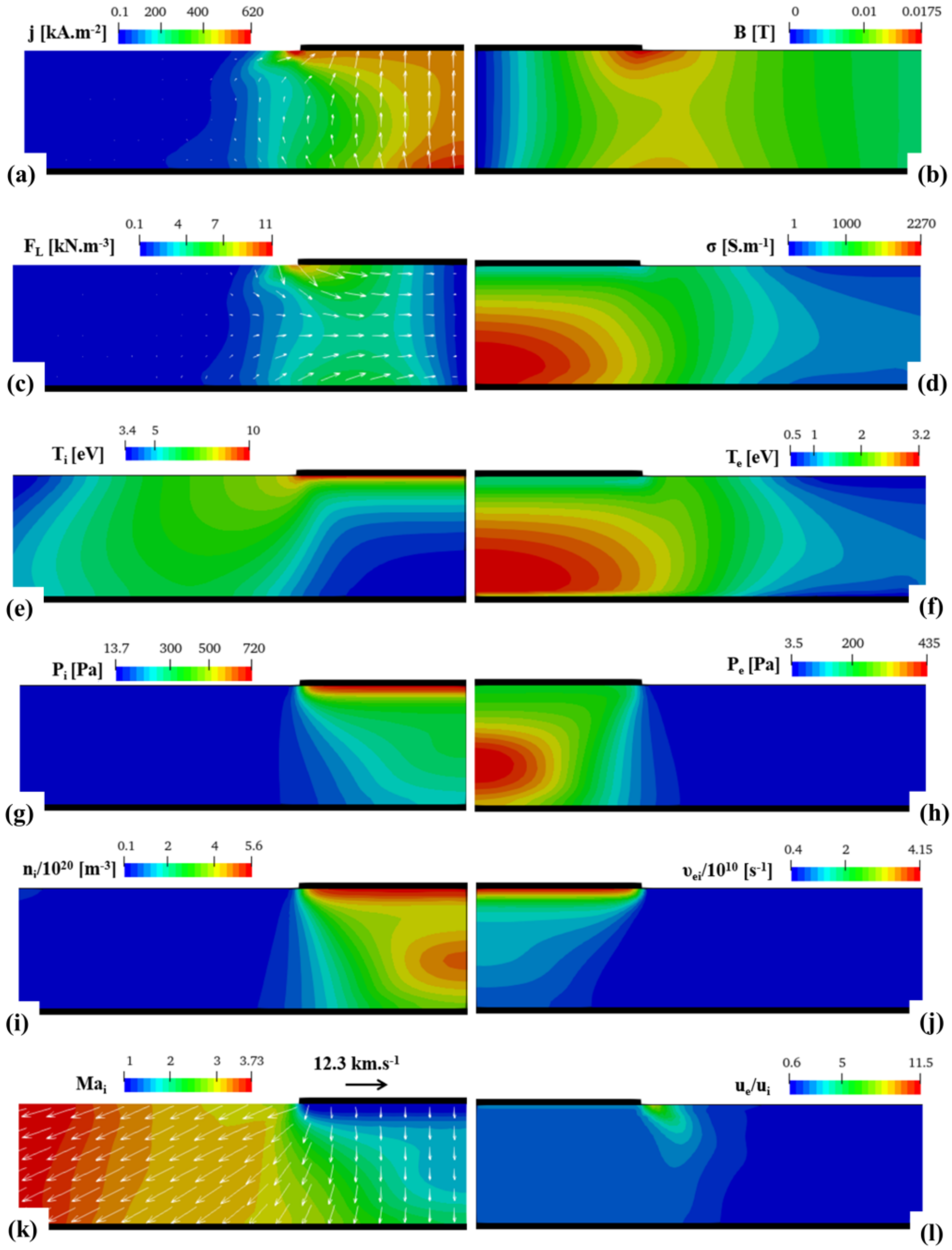

Figure S3: Field structures considering far-field assigned pressure of **30 pa** (a) Electric current density, (b) Magnetic flux density, (c) Lorentz force, (d) Electrical conductivity, (e) Ion temperature, (f) Electron temperature, (g) Ion pressure, (h) Electron pressure, (i) Ion number density, (j) Collision frequency, (k) Ion Mach number, (l) ratio of electron to ion velocity.
