# Supplementary material for: Experimental and numerical investigations of arc plasma expansion in an industrial vacuum arc remelting (VAR) process
Source: Sci Rep. 2022 Nov 27;12:20405. doi: 10.1038/s41598-022-24595-7 (PMC9701783; doi:10.1038/s41598-022-24595-7)
Supplement: Supplementary file 1 — Supplementary Information. [file 41598_2022_24595_MOESM1_ESM.zip › SuppMaterial_revised/Legends_Sup_Videos.docx]

Videos S1: Cathode Spots on the surface of electrode.

Videos S2: Arc plasma.
